# Visual tools to assess the plausibility of algorithm-identified infectious disease clusters: an application to mumps data from the Netherlands dating from January 2009 to June 2016

**DOI:** 10.2807/1560-7917.ES.2019.24.12.1800331

**Published:** 2019-03-21

**Authors:** Loes Soetens, Jantien A. Backer, Susan Hahné, Rob van Binnendijk, Sigrid Gouma, Jacco Wallinga

**Affiliations:** 1 Centre for Infectious Disease Control, National Institute for Public Health and the Environment, Bilthoven, The Netherlands; 2 Medical Statistics, Department of Biomedical Data Sciences, Leiden University Medical Center, Leiden, The Netherlands; 3 Department of Microbiology, Perelman School of Medicine, University of Pennsylvania, Philadelphia, USA

**Keywords:** transmission, cluster identification, mumps, algorithms, phylogenetic analysis, plausibility assessment

## Abstract

**Introduction:**

With growing amounts of data available, identification of clusters of persons linked to each other by transmission of an infectious disease increasingly relies on automated algorithms. We propose cluster finding to be a two-step process: first, possible transmission clusters are identified using a cluster algorithm, second, the plausibility that the identified clusters represent genuine transmission clusters is evaluated.

**Aim:**

To introduce visual tools to assess automatically identified clusters.

**Methods:**

We developed tools to visualise: (i) clusters found in dimensions of time, geographical location and genetic data; (ii) nested sub-clusters within identified clusters; (iii) intra-cluster pairwise dissimilarities per dimension; (iv) intra-cluster correlation between dimensions. We applied our tools to notified mumps cases in the Netherlands with available disease onset date (January 2009 – June 2016), geographical information (location of residence), and pathogen sequence data (n = 112). We compared identified clusters to clusters reported by the Netherlands Early Warning Committee (NEWC).

**Results:**

We identified five mumps clusters. Three clusters were considered plausible. One was questionable because, in phylogenetic analysis, genetic sequences related to it segregated in two groups. One was implausible with no smaller nested clusters, high intra-cluster dissimilarities on all dimensions, and low intra-cluster correlation between dimensions. The NEWC reports concurred with our findings: the plausible/questionable clusters corresponded to reported outbreaks; the implausible cluster did not.

**Conclusion:**

Our tools for assessing automatically identified clusters allow outbreak investigators to rapidly spot plausible transmission clusters for mumps and other human-to-human transmissible diseases. This fast information processing potentially reduces workload.

## Introduction

Individual case data originating from routine infectious disease surveillance more and more also include genetic sequence information. With increasing availability of different types of data (e.g. geographical data, time, genetic sequence), each adding their own dimension, and quantities of data rising, transmission-cluster identification of infectious diseases progressively relies on automated algorithms. A transmission cluster can be defined as several cases of an infectious disease which are connected by transmission of this disease from one person to another. A transmission chain is then defined as a series of cases connected by transmission events. Much work has been done on developing algorithms to identify transmission clusters of cases using large datasets [1]. Existing algorithms focus on cluster identification in time [2-9], in space or space-time [10-12], in genetics [13-15], or by combining all three data dimensions [16-18].

A major challenge with clustering algorithms is to balance specificity and sensitivity. If an algorithm lacks specificity, it finds clusters of cases even though there are no transmission events that link them. If it lacks sensitivity, the algorithm does not find genuine transmission chains. To be on the safe side, most algorithms have a high sensitivity at the expense of specificity and as a result also identify clusters of cases that are not genuine transmission clusters. We therefore propose cluster detection using algorithms as a two-step process: (i) detecting possible clusters of infectious diseases with an algorithm and (ii) assessing the plausibility that an identified cluster represents a transmission cluster.

While there has been much work on the first step, little research attention has been paid to methods for improving the plausibility assessment. Currently, identified clusters are usually assessed by epidemiologists who assess information and verify it through communicating with the municipal health services (MHS). This can be quite labour intensive, especially if there are many identified clusters stretching across multiple regions. Only recently, a study has been published that introduced a framework for computing epidemiological concordance of microbial subtyping data of *Campylobacter jejuni* [19]. Epidemiological cluster cohesion is based on time, geographical location, and environmental source distances with adjustable weights. This method requires the computation of a disease specific source distance matrix, making it difficult to apply generically. To our knowledge no further tools are available for careful plausibility assessment of automatically detected clusters.

In order to develop such tools, general characteristics for discriminating transmission clusters from non-transmission clusters have to be identified. We propose to assess the variation of clusters in their time, geographical location and genetic profile. The variation on these dimensions can be visualised by projecting cases on an epidemic curve, map, and phylogenetic tree, respectively, as well as by estimating the relative distance between clustered cases on these respective dimensions and comparing the distance to the inter-case distances from non-clustered cases. It is assumed that clustered cases will have smaller inter-case distances on these respective dimensions than non-clustered cases. However, there are exceptions: an outbreak may show large variation in time between the occurrence of cases (single persistent source, e.g. typhoid [20]), large variation in geographical distances between cases (initial cases travel large distances, e.g. severe acute respiratory syndrome (SARS) [21]), or include large genetic sequence variation in the pathogen causing the outbreak (fast mutating strains, e.g. Ebola [22,23]). In order to settle several of these exceptions, intra-cluster correlation between the data dimensions time, geographical location and genetics can be used as another discriminatory characteristic. In genuine transmission clusters, variation on one dimension tends to be correlated with the other dimension. For example, with tuberculosis, cases within a genuine transmission cluster with a larger genetic distance, also tend to have a larger distance in time [24].

The largest hurdle to effectively use algorithms in outbreak investigations is the interpretation of their output, rather than the application of the algorithms themselves. As visualisation techniques support fast processing of large amounts of information, developing tools for visually assessing the plausibility of transmission clusters identified through statistical algorithms may help outbreak investigators [25]. Moreover if data are available in a timely fashion, this may allow pointing outbreak investigators to the most plausible signals first, which, when time is scarce, may facilitate task prioritisation. Finally, outbreak information, such as what is obtained with various available tools (e.g. typical cluster size, typical inter-case distance and correlate estimates between dimensions for a specific disease), might contribute to our current understanding of transmission model parameters [26,27].

To apply and assess the tools that we develop, we use mumps notification and sequence data reported between 2009 and mid-2016 in the Netherlands. We specifically chose mumps in the Netherlands as it has been intensively studied over the past few years, with comprehensive documentation available [28-33]. In the Netherlands, mumps is a notifiable disease and symptomatic cases are reported to the MHS by physicians and/or laboratories. Cases are either notified when there is a laboratory confirmation or when there is an established epidemiological link with a confirmed case. In case of laboratory confirmation, the national reference laboratory aims to obtain material from regional laboratories for further sequencing. Sequencing provides information on the circulating genotypes and helps to assess whether there is endemic circulation or new introductions of mumps viruses in the country. 

Currently, epidemiologists mainly rely on epidemic curves (time data dimension) to detect mumps outbreaks. We set out to assess whether combining geographical location, time and genetic information can contribute to mumps cluster identification. We use an existing clustering algorithm which can take into account these three data dimensions [16,17] (hereafter: the time-place-type clustering algorithm), and which is already in use to identify outbreaks of various diseases in the Netherlands, such as meningococcal W disease, meticillin-resistant *Staphylococcus aureus* (MRSA) [17] and echovirus type 6 [34]. We develop and validate visual tools to determine whether identified clusters with this algorithm represent transmission clusters. 

## Methods

### Data

In this study, we include all notified mumps cases in the Netherlands who were diagnosed between 1 January 2009 and 31 May 2016. Notification criteria for mumps include more than one related symptom (i.e. acute onset of painful swelling of the parotid or other salivary glands, orchitis, or meningitis) and laboratory confirmation of infection or an epidemiologic link to a laboratory-confirmed case. The notification criteria did not change during the study period. 

For our analysis, for each case we require data on three factors. The first is the disease onset date, which is collected during routine surveillance. The second is the geographical location. The geographical location can be any location that is most relevant for the transmission pattern of the disease under study. For pragmatic reasons, this is usually the location of residence of the case, but this might also be a working address or other place visited. In this study, we used more specifically the latitude and longitude of location of residence of the cases. In the Netherlands, cases’ four digit postal code of residence is collected during routine surveillance. We take the centroids of the four digit postal codes and use its latitude and longitude as the input variables for the algorithm. The third required factor consists of the sequences of the small hydrophobic (SH) gene (316 bp), the haemagglutinin/neuraminidase (HN) gene (1,749 bp), and the fusion (F) gene (1,617 bp). Sequences of these three genes are used in combination to distinguish between different mumps genotypes [35], and clusters within genotype G [33]. Since the algorithm that we use is only able to handle cases with data on all three dimensions, cases with missing data on one of the three required factors are excluded from our analysis. 

### Cluster algorithm

In order to find infectious disease transmission clusters using time, geographical location, and genetic information, Ypma et al. [16] have developed an algorithm to combine pairwise distances between cases on all three data dimensions into one metric. The algorithm sorts cases by relatedness on all three dimensions and subsequently defines a relative distance for all possible pairs of cases reflecting the number of cases found in between the two cases. The relative distances (dissimilarities) for each dimension are calculated, and the combined dissimilarity (*d_combi_*) between every pair of cases is then defined as the product of the separate dimension dissimilarities. Next, the cases are joined to form a hierarchical tree of related cases, based on *d_combi_*, using single-linkage clustering. For every cluster in the tree, statistical significance of each cluster given its height and cluster size is calculated using permutation. More details on the algorithm are presented in Supplement S1.

To demonstrate the tools in this paper, we choose a p value < 0.001 as cut-off level for significance of clusters and consider only the clusters that are not nested within other identified clusters (hereafter ‘highest unnested clusters’) at that cut-off level. Since the p value cut-off level is an arbitrary choice, we add flexibility to the tool, by allowing setting cut-offs for p value, maximum tree-height and maximum cluster size.

### Assessing the plausibility of clusters representing transmission events

We have developed four tools to assess the plausibility that clusters identified by the time-place-type clustering algorithm represent transmission events. Below we describe each of these four tools in detail.

#### Overview visualisation of the clusters in the time, geographical location and genetic dimensions

To assess the variation in time, geographical location and genetic profile of the identified clusters, we visualise the distribution over time by projecting clusters on an epidemic curve (a histogram showing the distribution of cases over time). The distribution across geographical location is visualised by projecting cases coloured according to their cluster membership on a proportional symbol map, in which the point size is proportional to the number of cases at that location. In the interactive version of the tool, this map is replaced by an interactive dot map, which allows for zooming. The distribution across the genetic dimension is visualised by projecting clusters on an arbitrarily rooted maximum likelihood phylogenetic tree. Only the significant highest unnested clusters are visualised, using different colours for every cluster.

#### Hierarchical clustering tree to visualise the nesting of clusters

Identified clusters can be nested within larger clusters. The structure of the nesting provides valuable information on the strength of the clusters, for example, a cluster that contains several significant clusters at a lower nesting level is stronger than a cluster with no significant clusters at a lower nesting level. Therefore, the structure of the nesting is visualised by providing a hierarchical clustering tree of related cases, a dendrogram, based on *d_combi_*. The significant highest unnested clusters are visualised by colouring the end-nodes, and all significant clusters are visualised using black dots at the significant internal nodes.

#### Intra-cluster pairwise dissimilarity per dimension

To determine the impact of each dimension on *d_combi_* (time, geographical location or genetics), we calculate for every significant highest unnested cluster the pairwise dissimilarities per dimension (time, geographical location, genetics, and combined). The pairwise dissimilarities are a measure for intra-cluster variance for the different dimensions. The median dissimilarity is defined as the median of the pairwise dissimilarities (*d_time_*, *d_geo_*, *d_gen_* and *d_combi_*) per cluster. We visualise these pairwise dissimilarities using notched boxplots [36]. In a notched box plot, the notches extend 1.58 x interquartile range (IQR) / sqrt(*n*), which roughly corresponds to a 95% tolerance interval (assuming a normal distribution). The notches are then used to compare medians, i.e. non-overlapping notches for two different dimensions suggest that the medians are significantly different.

#### Intra-cluster correlations between the different dimensions

We visualise the intra-cluster correlation of the pairwise dissimilarities between the different dimensions. The intra-cluster correlation provides information on the internal cohesion of a significant cluster, for example, if cases within a cluster are close in time (small *d_time_*), are also close in geographical space (small *d_geo_*). In addition, the intra-cluster correlation coefficient between the separate dimensions and the combined dimension informs us on the contribution of each dimension to the combined dimension. For every significant highest unnested cluster, we compute the Spearman rank correlation coefficient *(r*) between the pairwise dissimilarities of all dimensions and its p-value [37]. We visualise the strength and direction of the correlation coefficients per cluster using a matrix layout. In the interactive version of the tool, one can hover over the matrices to allow for the correlation coefficients and p-values to pop up.

### Epidemiological validation

We use epidemiological information to check the validity of the identified significant highest unnested clusters. The gold standard for confirming transmission links is the presence of an epidemiological link between cases. However, this information is only available for a very small subset of mumps cases and is only described in free text fields, which is difficult to analyse. We therefore use mumps outbreaks described in the reports of the Netherlands Early Warning Committee (NEWC) as gold-standard-identified clusters and assess whether these outbreaks correspond to clusters identified with the algorithm [16]. The clusters that do not correspond with the reported outbreaks are considered false positives. In addition, we check whether the identified clusters are described in the literature.

### Analysis with only two dimensions

Since the algorithm cannot handle missing data and since genetic data are often delayed or missing, in Supplement S2 we show results when using time and geographical location data only.

### Availability

All analyses are performed in R 3.4.3 [38]. We have developed an R package *ClusterViz* containing an R shiny app to allow users to interactively set parameter values such as cut-offs for p values, tree heights, and cluster sizes. The R package can be downloaded from a github page (https://github.com/lsoetens/ClusterViz). A demo file for testing the tool is also available with this package (as described below). Considering the genetic data used in this study, all F gene, SH gene and HN gene sequences are submitted to the GenBank database and are available with the accession numbers KJ125045–51, KJ125053–9, KJ125061–7, and KU756625-930. 

### Ethical statement

Due to privacy concerns, data on date of diagnosis and geographical location are not published in any public database. Thus we have slightly obfuscated the time and geographical location data and have added the data file as a demo file to the R package. 

In accordance with Dutch law, no informed consent was required for this study using anonymised routine surveillance data.

## Results

Between 1 January 2009 and 30 June 2016, 2,039 cases of mumps were reported in the Netherlands. A sequenced sample of the SH, HN, and F gene was available for 118 (5.8%) of the cases. Of the 118 cases with sequenced data, six had missing geographical data. Therefore, 112 (5.5%) cases were included in the analyses. These cases were mainly male (n = 65; 58.0%) and had a median age of 24 years (IQR: 20–27 years). In this study period, 14 mumps related signals were reported by the NEWC (Table 1).

**Table 1:** Summary of all mumps outbreak reports by the Netherlands Early Warning Committee between January 2009–May 2016 (n = 14)

No	Date reported	Reported by	Covering time period	Number of cases in report	Age range (years)	Remark/source	Cluster number according to current study
1	09 Apr	RIVM	Aug 2007–Apr 2009	171	NR	Start of nationwide mumps epidemic	4
2	12 Feb	RIVM	Dec 2009–Feb 2012	1,264	NR	Overview of nationwide mumps epidemic	NL
3	12 Apr	GGD Gelderland - Midden	Mar 2012	22	15–26	Party	5
4	12 Jul	GGD Hollands - Noorden	Jul 2012	3	6–8	School	NL
5	12 Aug	GGD Zaanstreek - Waterland	Jul 2012–Aug 2012	21	16–48	Unknown	5
6	13 Feb	Utrecht	Feb 2013	8	NR	Unknown	5
7	13 Jun	GGD Hollands Noorden	Jun 2013	11	23–29	Unknown	NL
8	13 Nov	GGD Zaanstreek - Waterland	Sep 2013–Nov 2013	16	4–47	All living in Volendam	3
9	13 Nov	GGD Groningen	Sep 2013–Nov 2013	13	17–36	Students	NL
10	14 Feb	GGD Zaanstreek - Waterland, GGD Haaglanden	Feb 2014	3	25–30	Work in healthcare setting	NL
11	15 Apr	GGD Haaglanden	Mar 2015–Apr 2015	5	NR	Sports club	2
12	15 Jun	GGD Haaglanden	Apr 2015–Jun 2015	NR	NR	Students linked to school and earlier cluster at sports club	2
13	16 Mar	GGD Brabant Zuidoost	Feb 2016–Mar 2016	6	18–40	Carnival	1
14	16 Apr	GGD Hart voor Brabant	Mar 2016–Apr 2016	6	17–23	Friends/party	1

GGD: Gemeentelijke GezondheidsDienst (Municipal Health Service); NL: no link to a cluster; NR: not reported; RIVM: Rijksinstituut voor Volksgezondheid en Milieu (National institute for public health and the environment).

**Figure 1:**
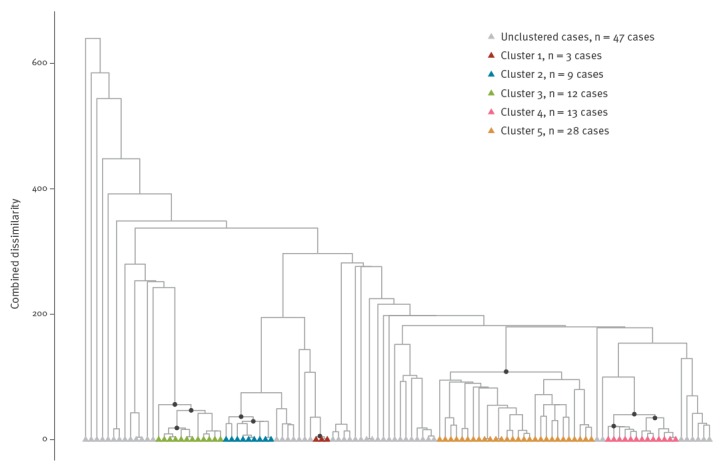
Hierarchical clustering tree of the combined dissimilarities of all dimensions^a^ for cases of mumps in the Netherlands, January 2009–May 2016 (n = 112 cases)

^a ^All dimensions include time, geographical location or genetic dimensions.

**Figure 2:**
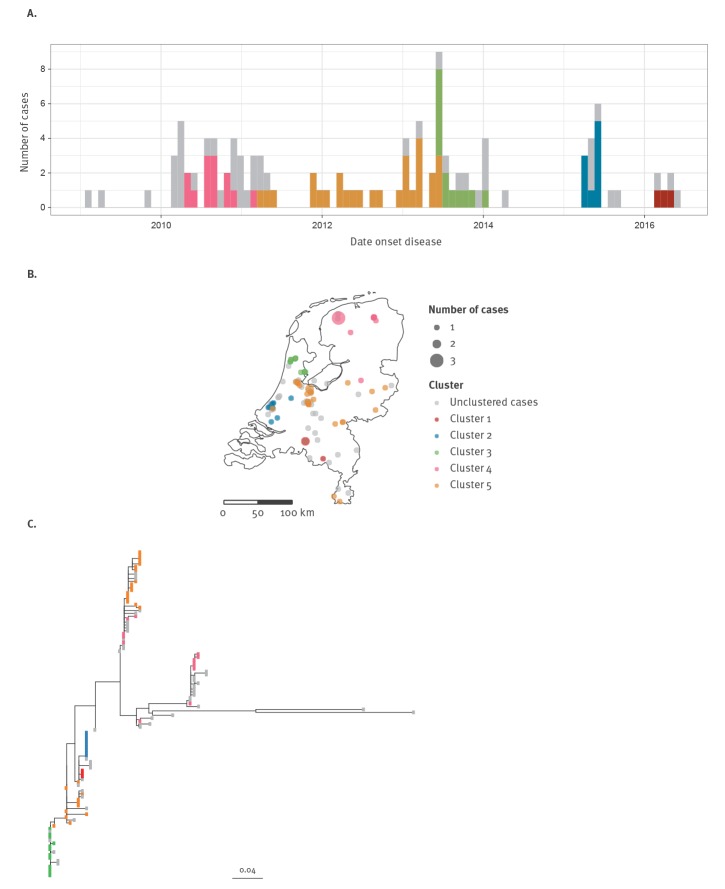
Identified clusters of mumps with cases projected on (a) an epicurve (time), (b) maps of the Netherlands (geographical location) and (c) an arbitrarily rooted maximum likelihood phylogenetic tree of the pathogen sequences (genetics), Netherlands, January 2009–May 2016 (n = 112 cases)

**Figure 3:**
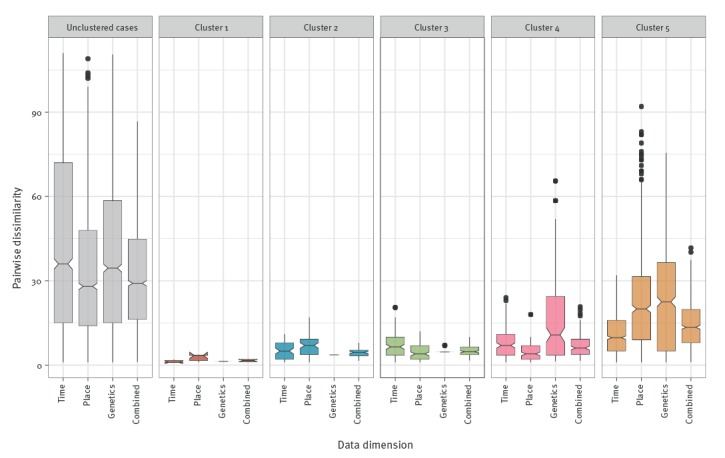
Notched boxplots of the pairwise dissimilarities per dimension and cluster of mumps cases, Netherlands, January 2009–May 2016 (n = 112 cases)

**Figure 4:**
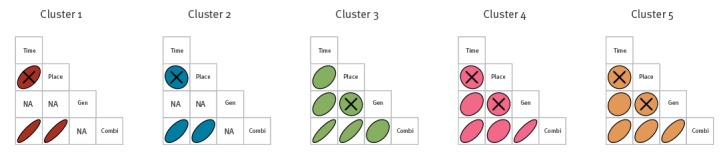
Matrix plots of the Spearman intra-cluster correlation between the pairwise dissimilarities of all four dimensions for cluster 1–5

Figures 1 to 4 represent output from our tool. The algorithm identifies 10 clusters with p < 0.001 of which five are nested (Figure 1). After collapsing the nested clusters into their parent clusters, five significant highest unnested clusters remain. Of those five highest unnested clusters clusters, cluster 2 (blue, n = 9), 3 (green, n = 12) and 4 (pink, n = 13) contain smaller clusters which are also significant, whereas cluster 1 (red, n = 3) and 5 (orange, n = 28) are not supported by other significant clusters at a lower nesting level.

The colours represent the cases belonging to significant highest unnested clusters. The black dots respresent significant clusters (p < 0.001) at all nesting levels.

To assess the plausibility of the clusters for a specific disease, we focus on the variation within clusters across the time, geographical location or genetic dimension. The clusters show differences in how the cases and samples are distributed over time (Figure 2a), geographical location (Figure 2b), and sequence space (Figure 2c). Compared with clusters 4 and 5, cluster 1, 2 and 3 are very compact on all three dimensions (time, geographical location, and genetics). While cluster 4 is relatively concentrated in time and geographical location, it is distributed across two branches of the phylogenetic tree. For mumps this makes it less plausible that all cases belong to the same transmission chain, as the mumps virus is characterised by a very low mutation rate [39]. For each of the two clusters nested within cluster 4 in the hierarchical tree, cases are located on two branches of the phylogenetic tree, suggesting that also the nested clusters contain substantial genetic disparity. Cluster 5 is quite dispersed on all three dimensions (time, geographical location, and genetics), making this cluster very implausible.

The colours indicate the significant highest unnested clusters identified with the time-place-type algorithm (cluster 1 is red, 2 blue, 3 green, 4 pink, 5 orange); the unclustered cases are depicted in grey. The scale represents the number of nucleotide substitutions per site.

We estimate and visualise for every significant highest unnested cluster the pairwise dissimilarities per data dimension (Figure 3). We find that the median pairwise dissimilarity is significantly lower on the combined dimension in all clusters when compared with the combined pairwise dissimilarity in the unclustered cases. Of the five clusters, cluster 1 has the lowest median pairwise dissimilarities on the three individual dimensions and their combination and cluster 5 has the highest intra-cluster variance on the three individual dimensions and their combination.

IQR: interquartile range.

For the unclustered cases (0) and for each cluster (1–5), the first column in each figure shows the pairwise dissimilarities in time, the second pairwise dissimilarities in geographical location, the third pairwise dissimilarities on genetics and the fourth shows the combined pairwise dissimilarity. The boxes’ height represents the IQRs. The horizontal lines in the notches represent the median of the pairwise dissimilarities and the lower and upper bounds of the notches can be interpreted as the 95% tolerance interval. The upper whisker extends from the box to the largest value no further than 1.5 * IQR from the box. The lower whisker extends from the box to the smallest value at most 1.5 * IQR of the box. Black dots indicate outliers.

We visualise the intra-cluster Spearman rank correlation coefficient (*r*) of the pairwise dissimilarities between the different dimensions (Figure 4). When looking at the correlation coefficients between the data dimensions time, geographical location and genetics, we can see that many correlation coefficients either cannot be estimated due to zero variance (identical sequences) on the genetics dimension (cluster 1 and 2) or are not statistically significant (p > 0.05). Only in cluster 3 the time dimension is significantly correlated with the geographical location (*r* = 0.4) and genetics (*r* = 0.5) data dimension, and in cluster 4 and 5 the time dimension is correlated with the genetics dimension only (*r* = 0.3 and *r* = 0.2 respectively). When then looking at the contribution of the individual data dimensions to the combined dimension, we can see that in cluster 1, 2 and 3, the dimension of time and geographical location contribute equally and strongly to the combined dimension (*r* = {0.9, 0.6, 0.9}), and in cluster 4 and 5 the dimension of genetics contributes the most information to the combined dimension (*r* = {0.8, 0.7}).

Combi: combined; gen: genetics; place: geographical location; NA: correlation cannot be estimated.

The width of the ellipse indicates the strength of the Spearman rank correlation coefficient *r*, while the slope of the ellipse indicates the direction of *r* (positive slope, positive *r*; negative slope, negative *r*). The crossed-out ellipses indicate insignificant correlations (p > 0.05), and an NA indicates a correlation could not be estimated due to zero variation on one or both dimensions.

As a measure of validity, we have assessed whether mumps outbreaks described in the reports of the NEWC correspond to clusters identified with the time-place-type algorithm. Clusters 1–4 are easily linkable to reported mumps outbreaks of the NEWC (Table 1). Given its time, period, and the spatial distribution, cluster 1 corresponds to outbreaks 13 and 14, cluster 2 corresponds to outbreaks 11 and 12, cluster 3 corresponds to outbreak 8, and cluster 4 corresponds to outbreak 1. Cluster 5 is the only identified cluster to which no clear reported outbreaks can be linked. Outbreaks 3, 5, and 6 might together possibly compose cluster 5. In addition to the NEWC reports, clusters 2 and 3 are described in references [33] and [32], respectively.

Finally, analysis using time and geographical location data only (Supplement S2) shows that our visual plausibility tools can also be used when data are only available for two dimensions. The cases included in the main analysis are representative for the total notified mumps cases from 2013 onwards, as the shape of the epidemic curves is comparable. However, before 2013 the shapes of the epicurves differ: in the main analysis the large peaks in 2010, 2011 and 2012 cannot be observed. In 2013–16, we identify six clusters using only two dimensions that are similar to those identified using three dimensions, we miss only three minor clusters. In the period before 2013, nine additional clusters are identified in the time-place analysis, of which three are very large (n > 40). The lesser plausible pink (cluster 4) and orange (cluster 5) clusters from the main analysis fall in the less representative period before 2013, so it might be due to unrepresentative sequencing in this period that transmission cluster detection with this algorithm is more difficult.

## Discussion

In this paper, we have introduced tools in order to assess the plausibility of transmission clusters. In the mumps case study, five significant clusters are identified, several of which also contain nested clusters. In assessing the plausibility of these significant clusters, the tools that we have developed all point in the same direction: clusters 1 (red), 2 (blue), and 3 (green) can be considered highly plausible; cluster 4 (pink) has moderate plausibility as the sequences related to it span across two branches of the phylogenetic tree; and cluster 5 (orange) has low plausibility. Compared with the other clusters, cluster 5 shows a relatively dispersed pattern across time, geographical location, and genetics; contains no nested clusters; shows relatively high intra-cluster dissimilarity on all dimensions; and shows the lowest intra-cluster correlation between all four dimensions. In our epidemiological validation, no clear reported outbreak can be linked to cluster 5. In contrast, the other four identified clusters are easily linkable to a reported outbreak.

The major advantage of our tools is that we use visualisation techniques to improve assessment of plausibility. Human vision supports fast processing of information [25], allowing for quick decision-making and this can therefore facilitate work for outbreak investigators. Besides fast processing, visualisation also allows for disease-specific characteristics in the assessment of the plausibility. While the first step in cluster detection, which identifies possible transmission clusters, can be done by algorithms, as it is a very generic process, the second step needs disease-specific considerations which cannot easily be incorporated in an algorithm. For example, in the case study our tools show that cluster 4 (pink) and cluster 5 (orange) span across multiple branches of the phylogenetic tree. A mumps expert knows that the mumps virus mutation rate is very low, which decreases the plausibility that these clusters represent unique transmission clusters.

An important aspect of our study is that only 5.5% of notified mumps cases had sufficient genetic information to be included. There are several reasons for this. First, mumps notification does not require laboratory confirmation in the Netherlands, but can also be based on the presence of an epidemiological link to a confirmed case. For these cases no material is available for testing and sequencing. Second, the obtained material is not always suitable for typing; viral loads can be low, which often result in failed sequencing. Third, we specifically chose to include only cases with an available sequenced sample of the SH, HN and F gene. Instead we could also have included cases with a sequenced sample of the SH gene only, as this would have resulted in a higher number of included cases. Nevertheless an earlier study [33] showed that the SH gene alone did not provide sufficient resolution for finding transmission clusters, whereas the combination of the three genes did. Since we aim to find transmission clusters here, including only sequenced samples of the SH gene was not an option. Because of these reasons, it is highly likely that the identified clusters in this study are actually larger or that some clusters are completely missed by the algorithm, as only one or two cases of a cluster might have a laboratory confirmation. This might explain the clusters reported by the NEWC (report 4, 7, 10 and 11), which were not identified by our tool (Table 1).

Our approach can handle incomplete data, such as cases with missing sequences, by performing a partial data analysis as in Supplement S2. Complete data analysis is shown in the main text. Further work can focus on extending the algorithm to allow for missing data on one or more dimensions. Especially considering that genetics information will often be missing if sequences are not available, one could replace sequence information by a categorical variable with pathogen subtype or other lower resolution indication of the pathogen type. Cases with a similar pathogen (sub)type would then have a distance of zero vs a distance of > 0 to cases with another (sub)type. The (sub)type information, however, should still have sufficient resolution to be able to contribute to transmission cluster detection. Similarly, if geographical location information is not available on the latitude/longitude level, one can think of lower resolution solutions. In this study, we use the centroids of the four digit postal codes as geographical location information. The information should have sufficient resolution to be informative. The tool is not limited by the number or type of dimensions. The addition or reduction of dimensions only requires small adaptions in our code and it is therefore straightforward to use our tools in combination with other algorithms, for example, space-time algorithms [10]. By increasing or decreasing the number of dimensions in the algorithm, the relative weight of the included dimensions decreases or increases as well, respectively. Depending on the quality of the data from the additional or removed sources, this may not be desirable. We have specifically chosen not to put weights on the separate dimensions, as determining the size of the weights is a very arbitrary decision. Instead, in the current study, we would rather interpret a cluster, which was primarily identified on the geographical location dimension, as less plausible, as the geographical location data are considered quite unreliable for mumps in the Netherlands. Indeed, information on place of residence (geographical location) is of questionable accuracy as mumps mainly occur among students who often have more than one living address (near the university and their parents’ address). It is then often not clear if the students actually live on the reported address at the time of the outbreak. On the other hand, if we would have had reliable geographical location information, other or more clusters might have been identified that now go undetected. Similarly, for mumps it is very unlikely that a transmission cluster is spread across multiple branches in the phylogenetic tree, so for mumps the genetic dimension might have more relevance than e.g. the geographical one. Instead of putting a weight on this dimension however, we considered cluster 4 and 5 as less plausible. Further work could investigate ways of determining the size of the weights, based either on the quality of the data (as discussed here) or on the type and transmission routes of the disease under investigation. A final issue regarding the internal correlation plots is that they are more useful when cluster sizes are larger. If the algorithm detects very small clusters (n < 4), we suggest to rely on the other tools to determine whether an identified cluster is plausible.

To conclude, our proposed tools for assessing plausibility of automatically identified clusters in time, geographical location and genetic dimensions can help outbreak investigators to focus on the most plausible clusters first. Timely availability of data are a prerequisite for this. In addition, using visual tools allow for fast and efficient information processing, which facilitates work. Mumps serves as an example in this study, but the algorithm can be transferred to other human-to-human transmissible diseases. 

## Supplementary Data

403000 Supplement S1

## Supplementary Data

470000 Supplement S2
