# Supplementary material for: Visual tools to assess the plausibility of algorithm-identified infectious disease clusters: an application to mumps data from the Netherlands dating from January 2009 to June 2016
Source: Euro Surveill. 2019 Mar 21;24(12):1800331. doi: 10.2807/1560-7917.ES.2019.24.12.1800331 (PMC6440581; doi:10.2807/1560-7917.ES.2019.24.12.1800331)
Supplement: Supplement S1 [file 1800331_SOETENS_SupplementS1.pdf]

S1 Algorithm to find infectious disease clusters using time, place and genetic information

This supplementary material is hosted by Eurosurveillance as supporting information alongside the article "Visual tools to assess the plausibility of algorithm-identified infectious disease clusters: an application to mumps data from the Netherlands dating from January 2009 to June 2016" on behalf of the authors who remain responsible for the accuracy and appropriateness of the content. The same standards for ethics, copyright, attributions and permissions as for the article apply. Eurosurveillance is not responsible for the maintenance of any links or email addresses provided therein.

In order to find infectious disease clusters using time, place, and genetic information, Ypma et al [1] have developed an algorithm to combine into one metric pairwise distances between cases on all three data dimensions. The algorithm sorts cases by relatedness on all three dimensions and subsequently defines a relative distance for all possible pairs of cases reflecting the number of cases found in between the two cases. For the time dimension (d_{time}), this is defined as the number of cases with a disease onset date between the disease onset dates of the two cases. For the place (d_{geo}) and genetic (d_{gen}) dimensions, cases are sorted by Euclidean distance and number of point mutations respectively to define pairwise relative distances. The relative distances (dissimilarities) for each dimension are calculated, the combined (time, place, genetic) dissimilarity (d_{combi}) between every pair of cases is then defined as the product of the separate dimension dissimilarities: $d_{combi} = d_{time} \times d_{geo} \times d_{gen}$. Next, the cases are joined to form a hierarchical tree of related cases, based on d_{combi}, using single-linkage clustering, i.e. a bottom-up approach that sequentially connects cases with the smallest d_{combi}. We use single-linkage clustering as it very well resembles the chain-like structure of transmission clusters [1]. The result of this step is a tree in which the height represents d_{combi}.

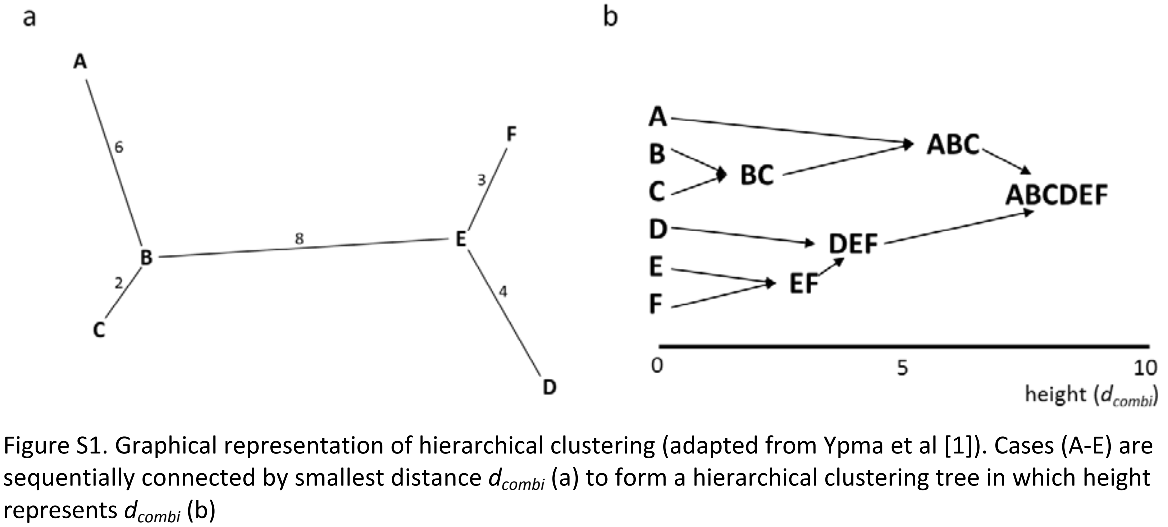

Figure S1. Graphical representation of hierarchical clustering (adapted from Ypma et al [1]). Cases (A-E) are sequentially connected by smallest distance d_{combi} (a) to form a hierarchical clustering tree in which height represents d_{combi} (b)

Next, for every cluster (i.e., dissection) in the tree, statistical significance of each cluster given its height and cluster size is calculated using permutation as follows: (i) the relative dissimilarities between pairs of cases in each dimension are permuted (random sampling without replacement), (ii) d_{combi} for every pair of combinations is computed, (iii) hierarchical clustering is repeated, (iv) for every cluster in the original tree, it is recorded whether a cluster of same height and size exists in the permuted tree, and (v) steps i to iv were repeated 10,000 times.

Finally, the p-value for each cluster is calculated as the number of times a cluster of the same height and at least the same size is found in the permuted trees divided by 10,000.

To demonstrate the tools in this paper, we choose a p-value < 0.001 as cut off level for significance of clusters and consider only the clusters that are not nested within other identified clusters (hereafter "highest unnested clusters") at that cut off level. However, the p-value cut off level is an arbitrary choice, and we can imagine that the users of the tool would like to experiment with other choices. We therefore add flexibility to the tool, by allowing setting cut-offs for p-value, maximum tree-height and maximum cluster size.

References

1. Ypma RJ, Donker T, van Ballegooijen WM, Wallinga J. Finding evidence for local transmission of contagious disease in molecular epidemiological datasets. PloS one. 2013;8(7):e69875
