# Supplementary material for: Visual tools to assess the plausibility of algorithm-identified infectious disease clusters: an application to mumps data from the Netherlands dating from January 2009 to June 2016
Source: Euro Surveill. 2019 Mar 21;24(12):1800331. doi: 10.2807/1560-7917.ES.2019.24.12.1800331 (PMC6440581; doi:10.2807/1560-7917.ES.2019.24.12.1800331)
Supplement: Supplement S2 [file 1800331_SOETENS_SupplementS2.pdf]

S2 Analysis with time and geographical location dimensions only

This supplementary material is hosted by Eurosurveillance as supporting information alongside the article "Visual tools to assess the plausibility of algorithm-identified infectious disease clusters: an application to mumps data from the Netherlands dating from January 2009 to June 2016" on behalf of the authors who remain responsible for the accuracy and appropriateness of the content. The same standards for ethics, copyright, attributions and permissions as for the article apply. Eurosurveillance is not responsible for the maintenance of any links or email addresses provided therein.

In the main paper we describe the analysis and results when including three data dimensions: time, place and genetic data. As genetic (sequence) information is often lagging behind or missing and to demonstrate the use of the algorithm with only two dimensions, we perform a similar analysis in this appendix with time and place data only.

We have made some minor adaptations to the algorithm: instead of multiplying with three dimensions, we multiply with only two dimensions.

Between January 1st 2009 and June 30th 2016 2,039 cases of mumps were notified in the Netherlands. Of those, 103 cases have missing information on location of residence and therefore 1,936 cases (94.9%) are included in this analysis (compared to 5.5% in the main article).

When repeating the analysis with similar settings as in the main article (p-value cut-off <0.001, clusters at highest nesting level), the results show only one large cluster encompassing almost all cases (n= 1,748, 90.3%). Plausibility of this cluster is very low given most indicators: it shows a very dispersed pattern across time and place, shows a high and almost indistinguishable intra-cluster variance on all dimensions compared to the unclustered cases, and shows no intra-cluster correlation between the time and geographic dimensions. Since this cluster has many underlying significant clusters at a lower nesting level, we decide to use different cut-off levels for the maximum tree height of the “highest unnested clusters”. We gradually lower this maximum height from 100% downwards, until we obtain clusters that are plausible considering our indicators. This is at a maximum tree height of 16% (height = 132) of the original tree height (height = 825), and these results are shown in Figure S1.

With these settings 17 possible transmission clusters are detected of various sizes (clustersize range: 6 -134). These 17 clusters are all quite plausible given most indicators, although the intra-cluster correlation coefficient between time and place dimensions is relative low or nonexistent for all clusters.

The identified clusters in the main article can also be observed in this analysis. The red cluster from the main article corresponds with cluster 3, blue with cluster 11, green with cluster 15, pink more or less with cluster 14 and orange more or less with cluster 16. The number of cases attributed to the clusters identified in the main article is larger. We notice that the lesser plausible clusters in the main article (pink and orange) do not agree completely to cluster 14 and 16 in this analysis. We can also notice that, when we compare the epidemic curve with the epidemic curve in the main article, from 2013 onwards the pattern is roughly similar, which might indicate fairly representative sequencing in that time period, but is quite different in the period before 2013. We can see some large peaks in 2010, 2011 and 2012, which are not depicted in the epidemic curve of the main article. The lesser plausible pink and orange cluster fall in this period before 2013, so it might be due to unrepresentative sequencing in this period that transmission cluster detection is difficult. This might also be the reason that the quite large (n> 40) clusters 12, 13 and 17 are not picked up in the main article.

With this additional study we show that it is quite well possible to use these plausibility tools with two dimensions only. We have also discovered that the cases included in the main analysis are quite representative for the total notified mumps cases from 2013 onwards, and that in this period similar clusters are detected if only two dimensions would have been used. We conclude that biased sequencing can influence cluster detection with this algorithm, so its therefore always important to aim for a representative sample of the total population for sequencing. In addition, this analysis also shows that even with a small representative sequenced sample of the total population, a great amount of cluster information is captured.

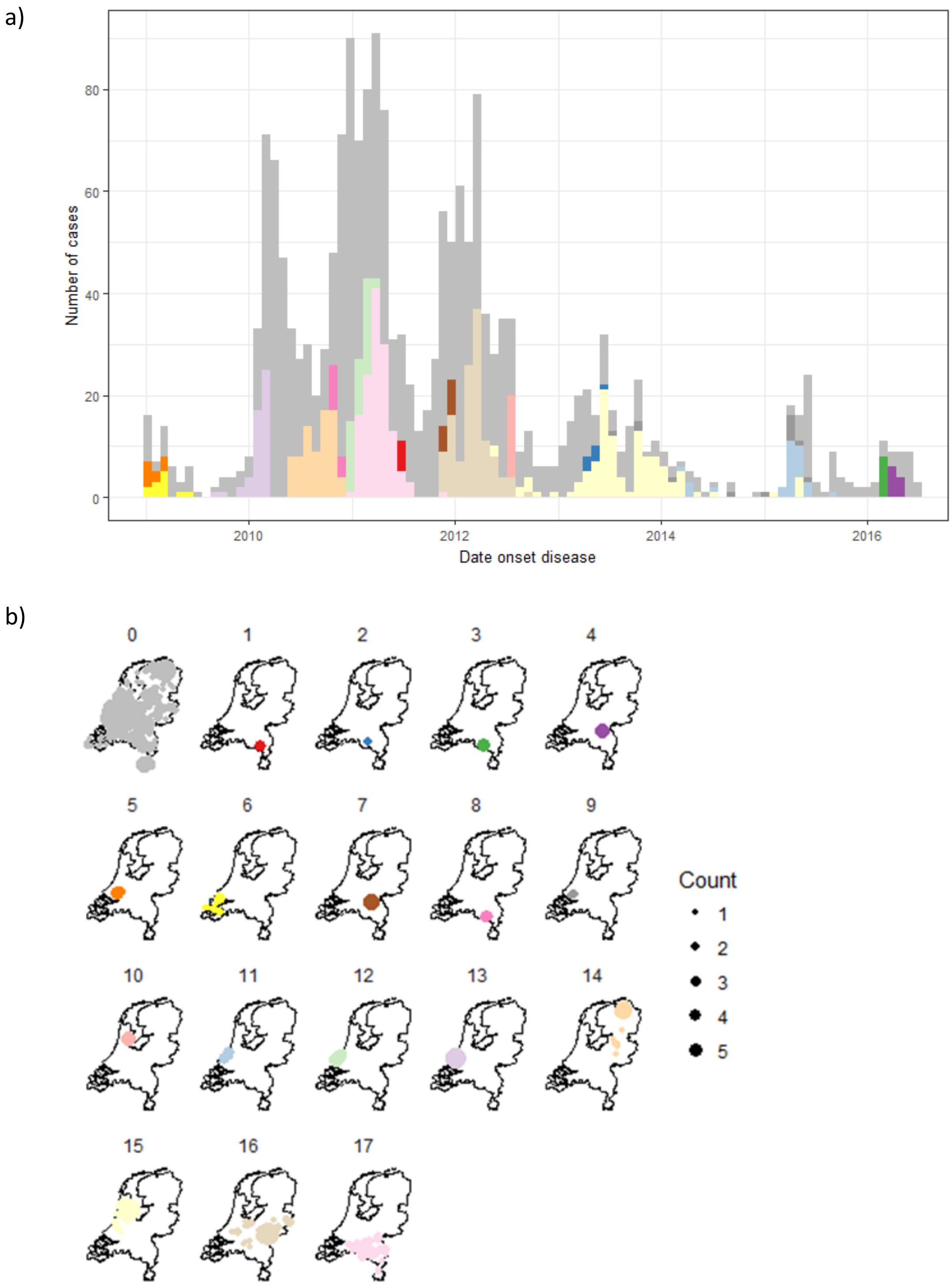
a)
Number of cases
0
20
40
60
80
2010
2012
2014
2016
Date onset disease
b)
0
1
2
3
4
5
6
7
8
9
10
11
12
13
14
15
16
17
Count
1
2
3
4
5

c)

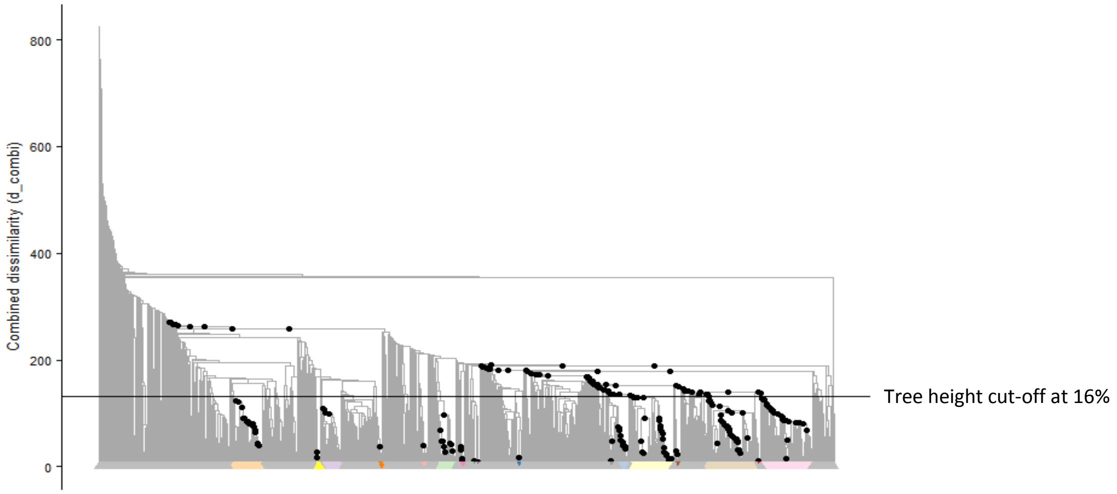

d)

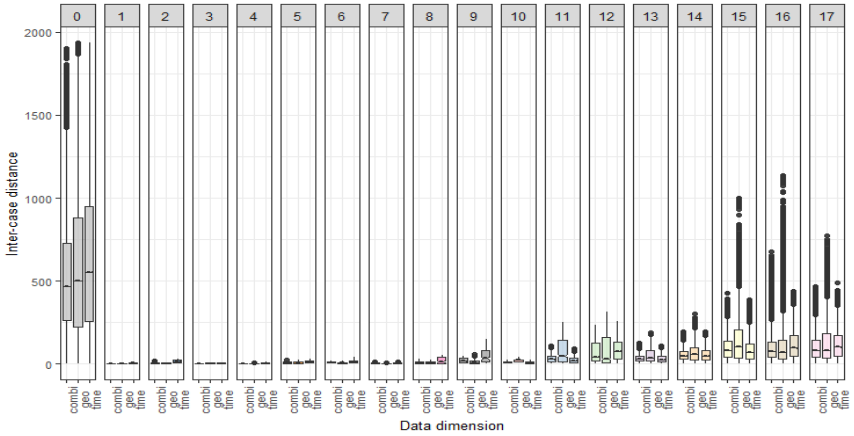

e)

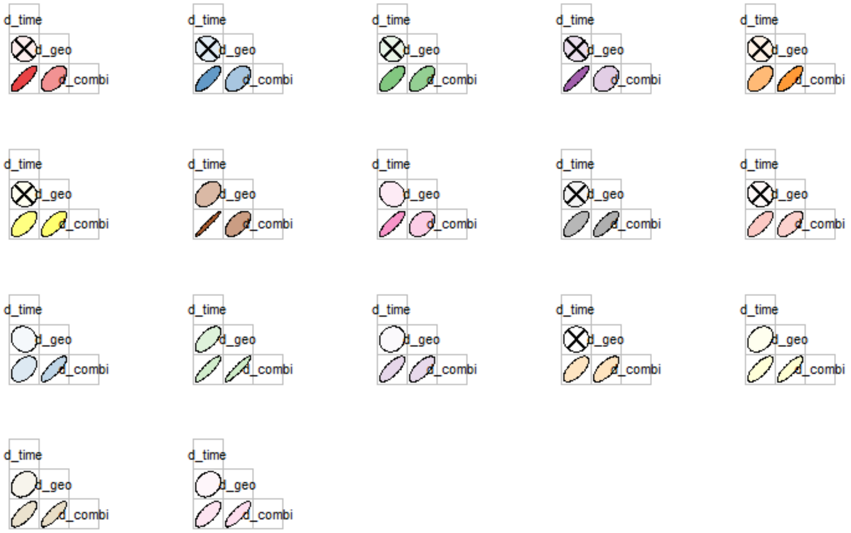

Figure S1. Results of the cluster analysis using a p-value cut-off of 0.001 and only considering the clusters at the highest nesting level with a maximum tree height of 16% of the total tree height. a) epidemic curve, b) map of unclustered and clustered cases, c) hierarchical clustering tree, d) notched boxplots of the inter-case distance per dimension and cluster, e) matrix plot of the Spearman intra-cluster correlation coefficients.
